# Thoroughly review the recent progresses in improving O/W interfacial properties of proteins through various strategies

**DOI:** 10.3389/fnut.2022.1043809

**Published:** 2022-10-26

**Authors:** Haozhen Zhang, Xue Zhao, Xing Chen, Xinglian Xu

**Affiliations:** ^1^Jiangsu Collaborative Innovation Center of Meat Production and Processing, Quality and Safety Control, Nanjing Agricultural University, Nanjing, China; ^2^Key Laboratory of Animal Products Processing, MOA, Nanjing Agricultural University, Nanjing, China; ^3^Key Lab of Meat Processing and Quality Control, MOE, Nanjing Agricultural University, Nanjing, China; ^4^College of Food Science and Technology, Nanjing Agricultural University, Nanjing, China; ^5^State Key Laboratory of Food Science and Technology, Jiangnan University, Wuxi, China; ^6^School of Food Science and Technology, Jiangnan University, Wuxi, China

**Keywords:** interfacial properties, protein emulsifier, oil in water emulsion, physical strategies, chemical strategies

## Abstract

Along with the future food market developing world widely, the personalized nutrition and rational function food design are found to be urgently attracted. Oil in a water (O/W) emulsion system has an excellent ability to maintain nutraceuticals and thus plays a promising role in producing future functional foods. Understanding the interfacial related mechanisms involved are essential for improving the quality of food products. Protein can effectively reduce interfacial tension and stable immiscible phases. The interfacial properties of proteins directly affect the emulsion qualities, which have gradually become a prospective topic. This review will first briefly discuss the interfacial-related fundamental factors of proteins. Next, the paper thoroughly overviewed current physical and chemical strategies tailored to improving the interfacial and emulsion properties of proteins. To be summarized, a higher flexibility could allow protein to be more easily unfolded and adsorbed onto the interface but could also possibly form a softer interfacial film. Several physical strategies, such as thermal, ultrasound and especially high-pressure homogenization are well applied to improve the interfacial properties. The interfacial behavior is also altered by various green chemical strategies, such as pH adjustment, covalent modification, and low molecular weight (LMW) surfactant addition. These strategies upgraded emulsion properties by increasing adsorption load, accelerating diffusion and adsorption rate, associated with lowering interfacial tension, and promoting interfacial protein interactions. Future researches targeted at elucidating interfacial-bulk protein interactions, unraveling interfacial behavior through *in silico* tools, exploring connection between interfacial-industrial processing properties, and clarifying the interfacial-sensory-digestive relationships of O/W emulsions is needed to develop emulsion applications.

## Introduction

Emulsion-based colloidal systems are ubiquitous in the food sector and show great potential in protecting and transporting nutraceuticals, enhancing the bioaccessibility of lipophilic bioactive substances and creating health-promoting functional foods (1). Along with the widespread development of the food market worldwide, personalized nutrition and functional food design are urgently needed; thus, emulsion-type food products attract increasing attention (2). It has been indicated that emulsion-type products are suitable for producing 3D print ink (3), biofilms (4), or personalized functional foods (5). Since the quality of emulsion-type products are closely related to emulsifiers’ interfacial behavior, in the food field, oil in water (O/W) emulsion-based interface science has been prospective and thriving in recent years.

The O/W emulsion is a metastable and thermodynamic system consisted with mainly three components, including oil, water and emulsifier. During emulsification, the lipid phase dispersed in an immiscible continuous aqueous phase driven by mechanical shear forces. To maintain the stability, it is necessary for emulsifiers to optimally cover the O/W interface and reduce the interfacial tension (6). As one kind of the major emulsifiers in food systems, proteins play a great role in stabilizing emulsion droplets by typically three steps: 1. diffusing from bulk aqueous toward the O/W interface; 2. after reaching the interface, proteins rearranging their native structure to orient the hydrophobic segments toward the non-aqueous phase, which was hidden inside the center of the coil structure; and 3. these adsorbed proteins tended to interact with neighboring molecules to form a quasi-two-dimensional network with elasticity and viscosity, which could prevent emulsion destabilization such as coalescence or flocculation afterward (7).

As mentioned, the formation of an interfacial layer depends on a complex phenomenon resulted from protein structural rearrangement, denaturation, disulfide bridge formation and intermolecular entanglement (8). The interaction among adsorbed protein layer, which also leads to a higher interfacial elastic modulus, is considered a predominant parameter in inhibiting coalescence and maintaining the stabilization of oil droplets (9, 10). One has concluded that the relationship between mesoscopic-level interfacial properties and macroscopic-level emulsion stability is not always straightforward (11). For example, no direct correlation was indicated between interfacial elasticity and emulsion stability (12). However, the interfacial behavior provides valuable information on emulsion quality. It should also be noted that both interfacial rheology and droplet interaction contributed to the bulk response (13).

**FIGURE 1:**
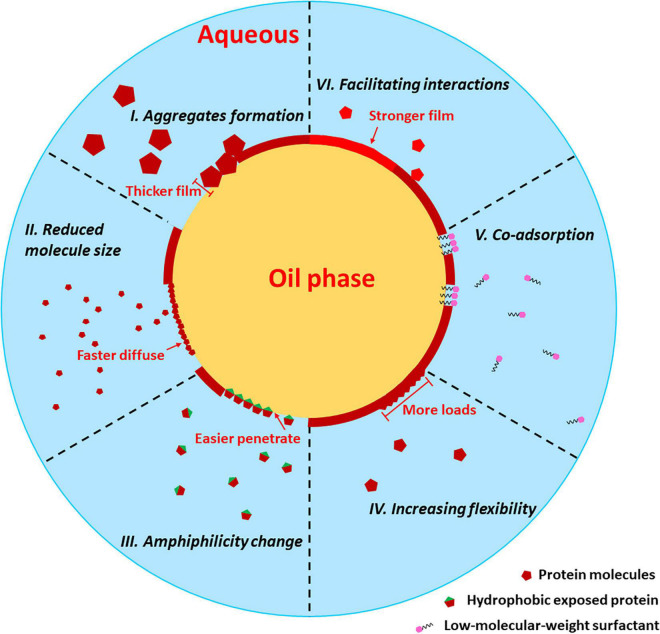
Potential pathways for various strategies to improve interfacial properties of protein.

Despite a few classic historical studies, this review mostly focused on works published in the recent 5 years, specifically concentrated on the single protein macromolecule tailored interfacial behaviors, which could be modified by low molecular weight (LWM) additives such as phenolic compounds, glucosamine, phospholipids, saponins or small peptides. Although the interface science is a hot topic in food field for recent years, many scientists focused on discussing the effects of a single processing on the interfacial behavior of protein. The comprehensive review and comparison of different treatments are still lacking. To the best of our knowledge, this is the first time that the protein characteristics, improvement strategies and the deep mechanisms are thoroughly reviewed and critically compared. In addition, the potential trends in the future are also raised with great cautions. Since multicomponent and particle-like emulsifiers have been fully reviewed recently (6, 14), to simplify the topic, the interfacial properties of Pickering dispersions and mixture macromolecular emulsifiers such as protein-polysaccharide or protein-protein complexes would not be discussed. In the present work, we introduced how protein features would affect its O/W interfacial and emulsion properties. Then, the physical treatments, including heating, high-pressure treatment, high-pressure homogenization, high-intensity ultrasound and others, for improving interfacial activities were thoroughly summarized. Furthermore, we highlighted the chemical-based application giving rise to promoting emulsion properties of protein. The potential mechanisms for the improvement are summarized and illustrated in Figure 1. Future research trends in O/W interface science in the food field are raised.

## Emulsion related characteristics of protein

The nature of biopolymer proteins dominates their interfacial fate and determines the interfacial film thickness, curvature, protein–protein interactions and thus rheological properties. Such interfacial behavior determines the emulsion properties such as oil droplet distribution, stability and destability, including gravitational-led separation, coalescence, Ostwald ripening, droplet aggregation, bridge and depletion flocculation (15). Here we discussed some protein related characteristics of protein about how they affect emulsion properties by modulating interfacial behaviors, such as amphiphilicity, flexibility, primary sequence, protein aggregation, and concentrations.

### Amphiphilicity

The reason why protein could work as a great emulsifier is because both polar and nonpolar amino acid residues distribution, giving protein an amphiphilic nature. The amphiphilicity, namely, the distribution of hydrophobic and hydrophilic residues, greatly affects the adsorption behavior of proteins: an overly high hydrophilicity leads to an insufficient driving force to overcome adsorption entropy, while an overly high hydrophobicity might lead to less surface activity (6). In other words, enough hydrophobic residues are important for protein to come together with oil phase and interact with each other to form strong interfacial film (16), while the number of hydrophilic residues determines the molecular forces between protein-water and steric hindrance within droplets (derived from dangling hydrophilic groups) (17). Briefly, a more balanced polar and nonpolar group distribution is favored for interfacial adsorption and emulsion stabilization afterward, which is determined by the protein sequence (18). Xiong et al. (19) verified the pea protein isolate subunits (i.e., isoforms of vicilins) well balanced with hydrophobic and hydrophilic groups displayed better interfacial activity. Also, after enhancing the amphiphilic balance of chicken liver protein with succinylation, the proteins exhibited better emulsion properties and resulted emulsions with lower particle sized droplets (20).

### Flexibility

Conformational flexibility refers to the ability for proteins to undergo conformational or even subunits rearrangement, which is negatively related to the hydrogen bonds between protein-water and the hydrophobic interactions and disulfide bonds among protein (21). It is considered to be a more important parameter than hydrophobicity in determining emulsion performance. Since flexibility is probably the most determinate characteristic influencing O/W interfacial properties, the structures of some typical proteins discussed in this work are depicted and listed in Table 1. The effects of flexibility of proteins on the interfacial properties highly depend on their native structure: (1) for globular protein, it is well acknowledged that globular proteins could rearrange in the interface and interact laterally to form a highly stable quasi-two-dimensional network (22). Many works performed in globular protein such as β-lactoglobulin (21), ovalbumin (23), and soy globulin proteins (24) have drawn a conclusion that an increased flexibility of globular protein leads to a higher rate for spreading at the biphase interface and faster adsorption, therefore favored emulsifying ability; (2) for flexible protein (i.e., β-casein), some classical works performed before have noticed a complete flexible conformation with low rigidity attributed to a thick but less dense interface film predominated by viscous property instead of elasticity, which negatively affected emulsion properties (25, 26). Unlike globular β-lactoglobulin, random coiled Na-casein forms a rather softer protective membrane, while the former is considered to form a cohesive and elastic matrix within the interface (27). To compare how conformational flexibility of protein impact interfacial behavior of β-lactoglobulin, the protein was treated with pH 7.0/pH 7.0+100 mM NaCl/pH 9 and their interfacial shear and dilatational properties were studied. This proved that at pH 9.0, the protein state in the bulk phase exhibited higher flexibility, therefore enhanced its rearranging and film forming ability by improving pronounced hydrophobic interactions and lowering interfacial molecular densities (28). Kieserling et al. (29) proved that the improved structural flexibility by using high hydrostatic pressure would increase the density of the interfacial film. Similarly, the higher molecular flexibility induced by phosphorylation and glycosylation also led to a much higher emulsion activity index (EAI) and emulsion stability index (ESI) owing to the larger adsorption ratio on the oil droplet surface (30, 31). Li et al. (24) established the correlation between molecular flexibility and emulsion ability as affected by the Maillard reaction and found that the correlation coefficient was relatively high (0.92). Accordingly, if the regular helix subunits dominate the secondary structure, the protein fraction shows a lower adsorption capacity (10). It has also been found that subunits of hexamer cruciferin protein with more hypervariable regions, extended loop, and more solvent-exposed surfaces would have better interface stability (32).

**TABLE 1:** Detailed information about globular and flexible structural proteins mentioned in this work.

No.	Protein name	Type	Mw (kDa)	Source	Structural properties in solution	3D coordinates (fetched by Pymol)	References
1	Lysozyme	Globular	14.3	Egg white protein	Small globular protein with four disulfide linkages. The proportions of SS at pH 7.0 are 36% α-helix, 15% β-sheet, 15% turn and 34% random coil.		Day et al. (33)
						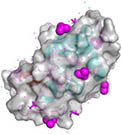 PDB ID: **2VBI**	
2	Bovine serum albumin	Globular	66.4	Bovine blood	Globular heat-shaped molecular obtained principally a-helix structure and included 3 homologous domains, which are divided into 9 loops by 17 disulfide bonds into 9 loops and each domain consisted of two subdomains.		Jahanban-Esfahlan and Panahi-Azar (118)
						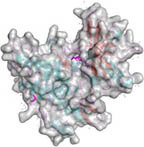 PDB ID: **3VO3**	
3	Myoglobin	Globular	17.9	Muscle tissue	Native myoglobin structure consists of 8 α-helix that are separated from each other by short loops.		Hou et al. (119)
						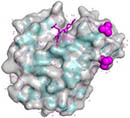 PDBID: **1A6M**	
4	Vicilin	Globular	50.8	Pea protein	Known as 7S, which belongs to three domain globulins, often existed in monocotyledonous and dicotyledonous plant species. Vicilins are always form trimers of a Mw of ∼150 kDa,		Xiong et al. (19)
						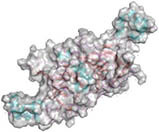 PDB ID: **6V7G**	
5	β-conglycinin α′-subunit	Globular	52.0	Soy protein	Three different β-conglycinin subunits are known as α′, α, and p, which shared similar primary sequence and are associated via hydrophobic interactions. One Cys residue possessed in α- and α′-subunit of β-conglycinin. The β-subunit generated to trimer and did not possess any cysteine residue.		Tandang-Silvas et al. (120)
						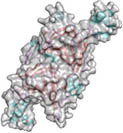 PDB ID: **1IPK** (*present 1 monomer instead of native homotrimers*)	
6	β-conglycinin α′-subunit	Globular	72.0	Soy protein			Tandang-Silvas et al. (120)
						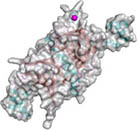 PDB ID: **1UIK** (*present 1 monomer instead of native homotrimers*)	
7	β-lactoglobulin	Globular	18.4	Bovine milk protein	Small globular proteins, the core of which has 5 cysteines forming 2 disulfide linkages and comprises 9 β-strands, a three-turn a-helix and a large internal hydrophobic pocket.		Jameson et al. (121)
						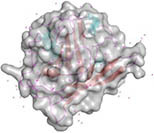 PDB ID: **3BLG**	
8	Ovalbumin	Globular	45.0	Egg white protein	One of the major constituent of egg-white protein belonged to serpin superfamily, consisting 9 α- helix and 3β-sheet.		Bhattacharya and Mukhopadhyay (122)
						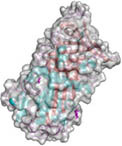 PDB ID: **1OVA** (*Chain A as chosen to present*)	
9	a-lactalbumin	Globular	14.2	Milk protein	Small globular proteins which contains 8 Cys forming 4 disulfide linkages with one large domain of three a-helices and one subdomain of three small antiparallel p-sheets structure. Two disulfide bridges connected the α-domain and β- subdomains and the loop is formed by one of the two bridges.		Mohammadi and Moeeni (123)
						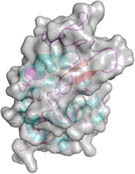 PBD ID: **1HFZ** (*Chain A was chosen to present*)	
10	β-casein	Flexible	24.0	Mil Protein	Usually a single form with 5 phosphates, contained mainly random coiled structure with no disulfide bond. β-casein shows the presence of approximately 5.5% α-helix, 34.5% β-sheet, 15% turns, and 44.5% unordered structure		Wong et al. (124); Cao et al. (125)
						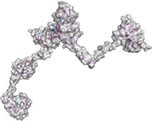 Homologous derive by I-TASSER	

The structure of all the proteins except for p-casein were obtained from RCSB PDB (https://www.rcsb.org/) with labeled ID code as cited from previous references. Since the structure of p-casein was not identified and recorded in PDB database, the I-TASSER server (117) was used to homologous its structure. The sequence of p-casein was from NCBI database (GenBank: AAA30431.1). The model with relative higher confident score and RMSD (C-score = -3.62, RMSD = 14.3 A) was chosen to exhibit. All the structures were captured by Pymol shown with surface (set as 20% transparency) and cartoon (reflecting secondary structure: cyan-helix, red-sheet, magenta-loop).

### Primary sequence

The primary sequence not only affects interfacial activity by posing different advanced structures but also directly changes protein interfacial performance. For example, a long hydrophilic or hydrophobic fragment could poorly affect interfacial activity of protein (19). Bovine serum albumin (BSA) and lysozyme are both globular-like proteins share high homology similarity, yet the former exhibited better emulsion performance (33). The main reason for this difference is credited to the varied distribution of cysteine residues. The cysteine residues in BSA are distributed in neighbor, while in lysozyme, they are located at the beginning and end of the sequence. Therefore, during adsorption, lysozyme exhibited higher internal cohesion and therefore a lower emulsion ability. Similarly, although monomeric human serum albumin (HAS) and BSA shared comparable structures in the bulk phase, because of different oligomerization stabilities, BSA was found to be more easily expanded and rearranged in the interface than HSA (18). Lajnaf et al. (34) ascribed higher efficiency in reducing the surface tension of camel β-casein than bovine β-casein to the higher content of Ile in the primary sequence, which bringing in more methyl group and thus increased hydrophobicity. Because of varied sequence, whey and casein protein prone to forming disulfide linked and hydrophobicity mediated aggregates, respectively, which resulted in different adsorption behavior (35). The primary sequence also determined the electrical charges and partitioning of peptides from cod bone, therefore causing different interfacial behavior and electrostatic repulsion forces between emulsion droplets (36).

### Aggregation level

Many works have been conducted recently to reveal the relationship between protein aggregation level and emulsion ability. As summarized by these studies, the aggregation-induced interfacial behavior changes are controversial. Pea protein obtained a much higher necessary protein concentration and longer adsorption time than whey protein, which is attributed to its larger molecular weight and supramolecular structures (37). For whey protein isolates, a high level of denaturation and aggregation in bulk phase leaded to a wider level of droplet distribution but showed minor effects on the diffusion rate. A combination of native aggregated whey protein isolate (WPI) resulted in the best interfacial performance, since native WPI dominated the interfacial adsorption process, while aggregated WPI could facilitate the viscosity of the continuous phase and inhibit phase separation (38). Zhou et al. (39) found the WPI exhibited better emulsion abilities than aggregates because of the interfacial film formed with denser and more brittle (quasi-) 2d structure, which could improve interfacial compactness by Marangoni-like effect. In contrast, soy β-conglycinin aggregates induced by ethanol treatment exhibited greatly improved emulsion performance compared to native protein due to the facilitated formation of bridged emulsions (40). Similarly, by forming soluble aggregates as induced by high pressure homogenization and heating, kidney bean protein obtained much higher emulsifying ability and activity (41). The predominant state of β-lactoglobulin (dimer or monomer) under various pH crucially affects the interfacial properties by changing phenolics binding location and thus altering the interfacial partition of protein (42).

### Protein concentration

The emulsion and interfacial properties of proteins are highly concentration dependent (39). A change in surface hydrophobicity and interfacial behavior (saturation level, adsorption rate, surface tension, interface denaturation, and diffusion) often emerges as a function of protein concentration (43). As reported, at lower protein concentrations (<1%), the protein adsorption toward O/W interface showed diffusion-controlled manner, while at higher concentrations (1–2%) protein adsorption was only diffusion-dependent because of the prevention of protein migration (44). A low protein concentration always led to flocculation due to an incompletely covered droplet surface, and the threshold concentration for stabilizing an emulsion was dependent on the protein species. Dridi et al. (27) mentioned that the interfacial properties are dependent on protein concentration at rather lower protein content and insensitive to agitation conditions because of limited coalescence process, while it is more reliable on emulsification power under high protein content. The protein concentration affects the interfacial protein profile of pea protein isolates. At a rather low concentration, the contents of pea protein adsorbed in the interface were ranked by aggregates > vicilin > legumin > convicillin, while at saturated adsorption, the protein content was ranked by vicilin > legumin > aggregates > convicillin (45). By using a novel microfluidic device, it is checked at a low protein concentration that the adsorption time and interface are vital to coalescence stability (46).

### Physical and chemical strategies targeted at improving the protein interfacial properties

The protein species and corresponding parameters for all the physical and chemical treatments used for improving the interfacial properties of proteins are summarized in Tables 2, 3, respectively.

**TABLE 2:** Recent studies concentrated on physical treatment extensively applied to improve the interfacial and emulsion properties of proteins.

No.	Protein species	Treatment	Parameters	Main findings	Reference
1	Soy protein isolates	Heating	90 and 120 °C for15 min	Heating increased the ratio of adsorbed protein on the interface layer and decreased the size of oil droplets.	Li et al. (78)
2	Pea protein	Heating	95°C for30 min	The heat treatment leaded to protein aggregation, increased the protein adsorption percentage and changes the protein profiles of interface layer.	Peng et al. (50)
3	Whey protein	Heating	Acid-thermal treatment: heated at 85°C for 24 h at pH 2.0 (nanofibril); Neutral-thermal treatment: heated at	The study fabricated and characterized native, nanofibril and nanoparticle whey protein. The high surface hydrophobicity promoted the adsorption of flexible nanofibrils on the interface. Interfacial tension was ordered as following: nanofibril > native > nanoparticles at pH 7.0.	Fan et al. (51)
			85 °C for 30 min at pH 6.0 (nanoparticle)		
4	β−lactoglobulin	HPT	100, 200, 300, 400, 500, and 600 MPa for10 minat20°C	The migration speed of the protein towards O/W interface was minor decreased only after 600 MPa HPT, but the adsorption rate was significantly increased after above 300 MPa HPT because ofexposed hydrophobic groups.	Kieserling et al. (29)
5	Whey protein	Heating. Mechanical treatment, HPT	80°C for 30 min; Rotor/Stator + Sonication; 16.3, 100, 300 MPa	Heating and 350 MPa HPT leaded to high level of hydrophobic group exposure and aggregates formation, whereas mechanical treatment only resulted in slight changes. Aggregation formation increased adsorb time at the interface but decreased reorganization period.	Moussier et al. (56)
6	Pea protein	HPT	200, 400, and 600 MPa	HPT showed more effects on emulsion properties of pea protein isolate at pH 3.0 and 5.0 by inducing open protein structure. At pH 7.0, protein exhibited optimal conformational properties for forming emulsion therefore HPT generate only marginal improvement on oil droplet size.	Chao et al. (57)
7	Soy protein isolates	HPT combined with pH-shifting	0.1,200,and400 MPa	The combination treatment of HPT and pH-shifting allowed the large aggregates in soy proteins unfolded and disassociated to form small soluble aggregates, thus favored emulsion formation.	Tan et al. (53)
8	Egg yolk protein	HPT with ultrafiltration	400 MPa for 5 min	The insoluble fraction in egg yolk protein could be transferred to soluble plasma fraction by HPT-ultrafiltration treatment, leading to emulsions with higher resistance to flocculation and higher stability.	Giarratano et al. (55)
9	Scallop protein	HPH	0, 20, 40, 60, 80, and 100 MPa	HPH treatment effectively increased ratio of adsorbed protein and emulsion stability from 20 to 100 MPa by facilitating aggregating. More protein mightily needs to fully cover interface after aggregates formation.	Wu et al. (61)
10	Chicken liver protein	HPH	0, 20, 40, and 60 MPa	The EAI and ESI showed highest value after treated by 40 MPa HPH, which is attributed to moderately exposed hydrophobic residues and spatial structure destruction.	Xiong et al. (126)
11	Oyster protein	HPH	20, 60, and 100 MPa	HPH treatment improved the EAI and ESI of oyster protein by increasing solubility to aggregate at the O/W interface and reducing the surface tension of the protein. The EAI showed highest value after 20 MPa treatment while the ESI reached highest after 60 MPa treatment.	Liu et al. (127)
12	Cod protein	HPH	20, 40, 60, 80, and 100 MPa	The interfacial adsorption protein percentage and shear storage modulus increased along with pressure increasing from 20 to 100 MPa, which is likely due to improved interfacial ability resulted by higher hydrophobicity and reduced particle size.	Ma et al. (62)
13	Whey protein and TGase-linked whey protein	HPH	30, 60, 90, and 120 MPa	Higher pressure of HPH leaded to better EAI for both whey protein and TGase-linked whey protein. TGase cross-linking probably blocked association of protein and oil therefore decreased EAI value. The emulsion properties is related to surface hydrophobicity, particle size and solubility.	Shi et al. (63)
14	Kidney bean protein	HPH	30, 60, 90, and 120 MPa	Thermally aggregated protein could be changes by HPH. Under 30-60 MPa, the soluble protein aggregates formed, which attributed to better emulsion properties, while the large aggregates were disrupted under 90-120 MPa, which reduced interfacial activity.	Guo et al. (41)
15	Faba bean protein	HPH	15000 and 30000 psi (103, 207MPa)	The HPH treated protein showed decreased emulsion properties because the soluble supramolecular aggregates induced by HPH might compete with protein molecule at the surface thus impaired the viscoelasticity of interfacial layer.	Yang et al. (64)
16	Pea protein aggregates	Heating; HPF	heating to 90 °C; 70 and 130 MPa	Heating could allow better O/W interface and smaller droplets formation by promoting aggregates formation. The HPF decreased particle size and hydrophobicity of thermal- induced aggregates and higher pressure showed more positive effects on emulsion stability by inhibiting flocculation and creaming.	Oliete et al. (65)
17	β−lactoglobulin	HPH	100 MPa/4 cycles and 300MPa/5 cycles	Stronger HPH-modified protein displayed faster adsorption rate and quicker formation of viscoelastic interfacial layer by increasing surface hydrophobicity, while weaker HPH had litter impact on structural and interfacial properties on p-lactoglobulin.	Ali et al. (66)
18	Canola protein isolate	HIU	40 kHz for 15 and 30min	Ultrasound exposure time and pH co-determined protein denaturation, more disordered structure formation, protein particles destruction and aggregates dissociation. Therefore, the EAI values are increased at the pH of 4, 8 and 10, and the ESI values are improved at the pH of6and10.	Flores-Jiménez et al. (69)
19	Fish myosin	Arg-assisted HIU	Adding 40 mM Arg, 20 kHz for 3 min with 5, 10, and 20% amplitude	Both ultrasound and Arg addition synergistically favored adsorption capacity of myosin on the O/W interface. The 20% amplitude + 40 mM Arg group exhibited highest adsorption ratio, most rapid adsorption and equilibrium, and highest interfacial pressure after interfacial loading.	Shi et al. (70)
20	Whey protein isolate	HPH, HIU	HPH (0, 60, 90, and 120 MPa, 3 cycles), HIU(20 kHz, 120/360/600 W, 30 min)	The combination ofHPH and HIU treatment could increase the EAI and ESI ofwhey protein further than the individual treatments by changing the flexibility ofprotein thus hindering oil from re-aggregation.	Shi et al. (72)
21	Soybean protein isolate	HIU-Acid/alkaline treatment	pH3.0-200W20kHz(0,2,5,10,20 and 30 min)-pH7.0	Acid-HIU treatment would make the large soy protein aggregates to dissociate and unfold to form small soluble aggregates. A suitable time of HIU (10 min) leaded to a faster adsorption rate on the O/W interface, thus more effectively decreased the interfacial tension and allowed smaller emulsion droplets formation.	Huang et al. (71)
22	Whey protein isolate	Cold plasma	40 and 50 Wfor0,10,20,30, and40s	The cold plasma treatment could improve the interfacial activity of whey protein, as reflected by increased adsorption rate and lowered interfacial tension compared to untreated protein. This is likely due to changes of protein structure and/or introduced new groups leaded by plasma treatment.	Gong et al. (73)
23	Peanut protein isolate	Extrusion cooking	Extrusion zone I for 25°C, zone II for 50°C, zone III for 90°C, zone IV for 100/130/160°C	The insoluble protein particles in peanut protein isolate was changed to soluble active sample during extrusion cooking because of enzyme hydrolyzation by endogenous protease. Therefore the saturation surface load was expanded thus generated smaller emulsion droplets.	Chen et al. (75)

**TABLE 3:** Recent studies concentrated on chemical treatment extensively applied to improve the interfacial and emulsion properties of proteins.

No.	Protein species	Treatment	Parameters	Main findings	References
1	Chickpea protein	pH adjusting	Adjusting pH to 2.5, 5.0 and 7.5	Compared to pH 5.0 and 7.5, chickpea protein at pH 2.5 prior to the adsorption obtained highest interfacial viscoelasticity and thus better emulsion stability because of increased repulsion interactions and protein unfolding.	Felix et al. (13)
2	Faba bean protein	pH adjusting	Adjusting pH to 3.0, 5.0 and 8.0	The dilatational rheology measurements are less sensitive to pH than shear viscoelastic measurements. Higher pH resulted in increased interfacial film strength, however, a lower pH leaded to a faster adsorption.	Felix et al. (9)
3	Poppy seed protein	pH adjusting	Adjusting pH to 3.0, 8.0 and 10.0	At lower pH, protein exhibited more openly stretched structure and resulted in increasing of emulsion activity. The most stable emulsion formed at pH 3.0 with more uniform distribution and smaller oil droplets.	Aslan Türker et al. (77)
4	Rapeseed protein isolate	pH adjusting	Adjusting pH to 3.0, 4.5 and 6.0	Isolation parameters affected emulsion stability of rapeseed protein isolate. For protein precipitated at pH 3.0, it obtained optimum emulsion stability at pH 6.0, while the protein precipitated at pH 6.0 would exhibit optimal emulsion stability at pH 3.0.	Ostbring et al. (128)
5	Egg white/yolk	pH adjusting	Adjusting pH to 5.0, 6.0, 7.0, 8.0, 9.0, and 10.0	The egg white and yolk proteins showed different response of emulsion properties to pH: the EAI is highest at pH 10.0 for egg white but at pH 7.0 for egg yolk. Increased emulsion activity closely correlated with smaller particle size and higher surface tension.	Li et al. (78)
6	β-lactoglobulin	pH adjusting	Adjusting pH to 7.0 and 9.0	Although p-lactoglobulin had higher electrostatic repulsions at pH 9.0, it reached lower adsorption rate and higher interfacial tension at pH 7.0 due to more exposed hydrophobic regions, which showed greater effect on adsorption behavior.	Schestkowa et al. (28)
7	Na-caseinate	pH adjusting	Acidification at pH 4.6 and 1.8 before and after emulsion process	No matter adjusting pH to 1.8 before or after emulsion, the Na-caseinate emulsion could keep stable from coalescence for at least 1 month. This is likely due to the protein expanded to the interface and increased monolayer thickness.	Dridi et al. (27)
8	β−conglycinin	pH adjusting	Adjusting pH to 3.0, 5.0, and 8.0	Compared to pH 5, protein can adsorb to the interface with higher adsorption kinetics at pH 3 and 8. Thus exhibited smaller droplet size. Near pI (pH 5) with low net charges would lead to large aggregation formation, which hindered the diffusion and rearrangement at the interface.	Tian et al. (79)
9	Microalgae protein	pH adjusting	Adjusting pH to 3.0, 5.0, and 9.0	The interfacial viscoelasticity modulus of untreated protein is highest near pI (pH 3 and 5), where proteins got lowest electrostatic repulsion and were able to aggregate and form strength intermolecular networks at the interface.	Dai et al. (81)
10	α-zein	Alkali-heat treatment	pH 11.5-70 °C 10 h-pH7.0	Alkali-heat treatment in ultrapure water environment effectively improved the emulsion properties of α-zein. This may resulted from increased molecular flexibility, which facilitated adsorption and unfolding of protein at the interface.	Dong et al. (82)
11	Soy glycinin	Acid treatment	pH 2.5-pH 7.0	Acid treatment caused improved emulsion stability and smaller oil droplets by increasing electrostatic repulsions between droplets and interfacial protein concentration.	Abirached et al. (84)
12	Chia protein	Alkaline treatment	pH 10 and 12-pH 4.5-pH 7.0	Compared to pH 12.0 treatment, the pH 10.0 treated sample exhibited better interfacial properties and retarded liquid drainage because of the formation of thicker surface film.	López et al. (83)
13	Canola protein isolate	Dephenol-Alkaline treatment	pH 11.0-pH 4.5- pH 7.0; pH 12.0-pH 7.0	The combination of dephenol and alkaline treatment promoted the solubility and emulsifying properties of canola protein isolate. Alkali-induced molten globular structure showed benefit effects on interfacial interactions in O/W interface.	Jiang et al. (85)
14	Whey protein	Phytic acid-protein conjugate formation	Conjugate phytic acid at different levels (0.01%, 0.05%, 0.1%, 0.15%, 0.2%, 0.25%)	The electrostatic bridge formed between cationic whey protein molecular and anionic phytic acid ions contributed to better emulsion stability, especially after adding 0.05% phytic acid. Phytic acid could prevent protein unfolding and/or aggregation at the O/W interface thus improve coalescence stability.	Pei et al. (129)
15	Whey protein	Protein-protein cross-linked by cinnamaldehyde	Oil phase added with different weight ratio of cinnamaldehyde (CA) and medium chain triglyceride (MCT): 10:0, 9:1, 7:3; 5:5; 3:7, 0:10	CA in the oil phase could effectively enhance protein-protein cross-linking formation in the O/W interface, as shown by increased surface load content and reduced interfacial tension.	Chen et al. (89)
16	Whey protein	Proanthocyanidin-protein conjugate formation	Concentration of proanthocyanidin is 0.01% or0.1%	The addition of polyphenols leaded to highly aggregated lipid droplets at pH 4 during storage, but not at pH 3, 6.5 or 8. This is due to the electrostatic repulsion between the lipid droplets are more reduced at pH 4.	Chen et al. (88)
17	Whey protein and silkworm pupae protein	Protein-protein cross-linked by cinnamaldehyde	Adding 2 wt.% cinnamaldehyde	For both proteins, use of cinnamaldehyde agent allowed the protein to form interfacial layers with stronger and more densely homogenous character according to dilatational and interfacial shear measurements.	Felix et al. (90)
18	Rice protein hydrolysates	Chlorogenic acid-protein conjugate formation	Adding 0-0.125% (w/v) chlorogenic acid under pH 9 condition	Chlorogenic acid covalently interacted with rice protein hydrolysates and improved the emulsion activity under 0.025% addition content. The reason included increased interfacial adsorption ratio and thicker interfacial film formation.	Pan et al. (91)
19	Flaxseed protein isolate	Flaxseed phenolic-protein conjugate formation	Adding 0.3 mM phenolic compounds under pH 9 condition in the presence of oxygen	The conjugate formation could only increase the diffusion rate at low protein concentration (0.1 mg/ml) but showed no effects on interfacial behavior at higher protein concentration (1-10 mg/ml). Phenolic-protein complex formation decreased emulsion stability and elasticity of surface film because of the reduced surface charge density induced by phenolics introduction.	Pham et al.. (92)
20	Faba bean protein	TGase treatment	Protein incubated with TGase for 60, 120, and 240 min.	TGase treated for 60 min could maintain the emulsion properties of faba bean protein by increasing net surface charge, while excessive treating time (120 and 240 min) leaded to unwanted structure changes and induced coalescence phenomenon.	Liu et al. (93)
21	Whey protein isolate	Enzymatic hydrolysis and TGase cross-linking	Firstly, hydrolyzed with trypsin and alcalase to obtain about 2%, 8% and 14% hydrolysis degree; secondly, incubated with TGase (10 U/g protein) at 37 °C for 4 h.	The native globular whey protein is more prone to adsorbing at the interface compares to hydrolyzed samples, exhibiting higher emulsifying activity. TGase- dominated cross-linking rescued partial emulsion ability lost because of hydrolysis.	Yu et al. (94)
22	Soy protein isolate	TGase-catalyzed glycosylation	Protein incubated with TGase (10 U/g protein) and glucosamine (3:1, protein: sugar) at 37 °C for 2 h	The TGase catalyzed glycosylation is able to open the native rigid structure, expose the internal hydrophobic groups and increase the flexibility of soy protein. Therefore, the emulsion ability and stability are effectively increased.	Zhang et al. (31)
23	Buckwheat protein isolates	HIU combined with glycosylation	Protein incubated with dextran at 70°C for 80 min and with ultrasonic intensity of544.59 W/m^2^.	The HIU assisted glycosylation treatment resulted in a better emulsion ability of proteins because of occupying smaller area at the interface, packing more closely and forming thicker interfacial film.	Xue et al. (96)
24	Na-caseinate	Hydrolyzed with the commercial enzyme	Hydrolyzed at 65 °C with the enzyme activity of 1 and 15 nkat/ml and the final degree of hydrolysis reaches between 0.1 and 8.5%.	A lower level of hydrolysis degree (2.2%) allowed a network-like supramolecular particles self-assembly by hydrophobic peptide, and increased the stability of emulsions by 400%. While the higher level of hydrolysis leaded to smaller and spherical-shaped supramolecular structures, which resulted in phase separation within minutes.	Ewert et al. (95)
25	Soy glycinin/P-conglycinin	LMW surfactant mixing	0.5% protein mixed with 0.05%-0.5% soyasaponin	High level of soyasaponin (0.25-0.5%) addition loosened and unfolded the structure of protein at the interface by synergistically lowering interfacial tension, leading to long-term stability till 42 days.	Zhu et al. (106); Zhu et al. (98)
26	Mussel water-soluble proteins	LMW surfactant mixing	Mixing lecithin with 0–2.0% concentrations	High level of lecithin (1.5-2.0%) degraded the emulsion properties because of competitive adsorption, while intermediate concentration (1.0%) leaded to increased percentage of adsorbed protein and highest emulsion stability.	Zou et al. (99)
27	Na-caseinate	LMW surfactant mixing	Mixing sucrose ester with 0–0.3% concentrations	The interfacial tension kept decreasing along with increasing level of sucrose ester (SE). Adding 0.01% SE sharply increased the surface dilatational modulus but higher level ofSE reduced the modulus.	Zhao et al. (100)
28	β−lactoglobulin	LMW surfactant mixing	Mixing *Quillaja* saponin with 0.005%wt concentrations	The interfacial behavior of protein was distinctively changed by the addition of saponin. Mixed interfacial layers of protein and saponin exhibited higher viscous modulus. The emulsion properties are highly dependent on the content of both protein and saponin.	Bottcher et al. (130)
29	Myofibrillar proteins	LMW surfactant mixing	Mixing zein hydrolysates (ZH) with 0–10 mg/ml	Addition of5 mg/ml ZH resulted in highest ESI and smallest droplet size by promoting the adsorption of protein. At this level, interfacial film could form more compact and massive structure	Li et al. (102)
30	Chickpea protein isolate	Ultrasound + LMW surfactant mixing	Ultrasonic mixture of chickpea protein and ginseng saponin (0.5%) at 600 W for 0, 5, 10, 15, 20, and 25 min	Ultrasound treatment facilitated the bind affinity of ginseng saponin towards protein and improved the interfacial adsorption and emulsion stability of protein- saponin complex, especially treated for 15 min. Combination with saponin would increase electrostatic repulsion to prevent droplets aggregation.	Xu et al. (107)
31	Pea protein	LMW surfactant mixing	Adding with 3 × 10^–4^ M *Quillaja* saponin	The mixture ofpea protein and saponin developed a weakened interface and decreased interfacial cohesion than either of the individual, reflected by lower interfacial storage modulus than loss modulus.	Reichert et al. (8)
32	Whey protein isolate	LMW surfactant mixing	Mixing soy lecithin with 0–2% concentrations	Addition of low level of soy lecithin (0.25–0.75%) decreased the viscoelastic properties of emulsion interface, while intermediate level of lecithin (0.5-1.0%) promoted the interaction between lecithin and protein in the adsorption layer.	Wang et al. (108)
33	Soy protein	LMW surfactant mixing	4%, 6, and 8% w/w protein mixed with 1% w/w monoglycride	Under a certain protein content, adding monoglycride significantly increased the emulsion properties of conglycinin but showed negative impact on reduced- glycinin soy protein, which is resulted from the competitive effect on the interfacial adsorption.	Li et al. (110)
34	Soy protein	LMW surfactant mixing	1% soy protein isolate and its hydrolysates mixed with/without 0.1% wt monoglycride	The native soy protein obtained good interfacial properties by forming solid-like interfacial layers. The hydrolysates exhibited fluid-like interfacial film because of the presence of small peptide and relatively less p-subunits and acidic subunits. Monoglycride could replace soy protein and reduced the amount of adsorbed protein at O/W interface.	Chen et al. (45)
35	Gelatin	LMW surfactant mixing	The 0.8% gelatin solution was mixed with soy lecithin at a final concentration of 2%.	Soy lecithin could synergistically adsorbed onto the interface of oil/water with gelatin to stabilize emulsion	Zhang et al. (109)
36	Carioca bean	Enzymatic hydrolysis	Hydrolyzed at pH 7.0 and 55°C to realize degree hydrolysate of 6% and 9	The hydrolysates showed higher emulsion ability than protein at all pH values. Also, hydrolysis effectively increased the final stability of emulsions.	Los et al. (131)
37	Sunflower protein	Sonication: 20/40 kHz, 220 W Enzymatic hydrolysis and ultrasound	for 15 min at 45°C; Hydrolyzation: using alcalase (3000 U/g protein) to realize degree hydrolysate of 6, 12%, 18, and 24%	Producing of peptides by hydrolyzation leaded to the loss of emulsion ability of proteins. Therefore, higher level of hydrolysis degree resulted in lower EAI and ESI. Although the peptide could more quickly diffuse and adsorb on the interface, but the efficacy of interfacial tension reduction is decreased.	Dabbour et al. (114)
38	Lysozyme and bovine serum albumin (BSA)	LMW surfactant mixing	Different concentrations of [C**12**mim]Br (from 5 × 10^–8^ M to 5 × 10^–5^ M) mixed with lysozyme (7*10^–7^M) and BSA(1.5*10^– 8^ M)	Lysozyme showed different response to LMW emulsifier [C**12**mim]Br at various content. The interfacial dilatational modulus of lysozyme would decrease while [C**12**mim]Br content increased, indicating a competitive adsorption. However, BSA and [C**12**mim]Br are more likely to synergistically adsorbed at the O/W interface because of structure unfolding.	Cao et al. (115)
39	Sunflower protein	Adding with phenolics	Mixing chlorogenic acid (CA, 0.1 wt%) with protein (0.1% wt) at different level (1:1 and 1:5, w/w) under pH 7.0	Non-covalent protein-CA complex readily adsorbed at the O/W interface and more effectively decreased the interfacial tension compared to solely protein. The phenol group could position at the interface and improve chemical stability of emulsion dispersed phase.	Karefyllakis et al. (116)
40	Ovalbumin	Wet-heating phosphorylation	Treated with sodium tripolyphosphate (4%, w/w) at 40°C for 0.25, 1, 2, 3, and 4 h	Medium-level phosphorylation showed highest emulsion ability, while the high- level group exhibited best emulsion stability. The introduction of phosphate group loosed the structure of protein and reduced the initial interfacial tension at O/W interface.	Tang et al. (30)

### Physical technologies

#### Thermal treatment

Thermal treatments have long been implemented in improving the emulsion properties of proteins through increasing interfacial adsorbed content and modifying advanced molecular structure (47). Particularly, it is shown that the preheating treatment could facilitate the emulsion activity of protein, which leading to smaller droplet size and lower interfacial tension of droplets (48). Besides emulsion activity, thermal treatment could enhance emulsion properties by leading protein aggregation. As suggested by Li et al. (49) and Peng et al. (50), although the increased molecular size potentially lowered the diffusion and rearrangement rate, it also allowed larger steric hindrance formation at the droplet surface, exhibiting higher interfacial viscoelasticity. Thermal treatment gave rise to the interfacial properties of plant derived protein because of thermally induced disulfide bond cross-linking, surface hydrophobic group exposure and especially protein aggregation (49). Similarly, heated pea proteins (95°C) exhibited a higher extent of protein aggregation and a much higher percentage of adsorbed protein at 0.1–0.5% protein concentrations (50). Thermal treatment also changed the interfacial properties through modifying the morphological state of the aggregates. By fabricating native whey proteins to form particle- and fibril-like aggregates, proteins exhibited effectively improved emulsion ability. The different morphologies also led to altered responses of the interfacial properties to pH (51).

#### High pressure treatment

As one of the most attractive non-thermal technologies, high-pressure treatment (HPT) (100–600 MPa) is often regarded as a cold pasteurization process, which is also capable to improve the interfacial properties of food proteins (52). The effective parameter of HPT varied as species-dependent manner. Some works suggested a ≥ 200 MPa pressure is available in improving interfacial properties, such as in soy protein isolates (400 MPa) (53), meat protein (200 MPa) (54), egg yolk protein (400 MPa) (55) and β-lactoglobulin (600 MPa) (29). However, in many cases, the increase in interfacial properties can only be observed in moderate pressure. For example, compared to 16 and 350 MPa-treated whey protein isolates, the 100 MPa pressurized protein exhibited better interfacial properties, reflected by the most closely packed interfacial film and highest interface protein loading content (56). One of the mechanisms for HPT in improving protein interfacial activities is by forming a pressure-induced molten globule state in the bulk water phase, which lagged the adsorption period but increased the affinity toward the hydrophobic oil phase (29). Yang et al. (1) considered the improvement is attributed to depolymerization of interfacial protein through HPT, while in egg yolk granule, the transformation from granule phosvitin to the soluble fraction favored the emulsion properties (55). To be addressed, the environment also decided the efficiency of HPT. For example, HPT (200–600 MPa) could only enhance the emulsion stability of pea protein isolates at pH 3.0 but merely affected the emulsion quality at pH 7.0 (57).

#### High pressure homogenization

High pressure homogenizer is usually consisted with a displacement pump and a homogenizer valve, which processed mixed dispersions to small droplets using high static pressure (10–100 MPa) (58). Although it is generally acknowledged that high-pressure homogenization (HPH) and high-pressure fluidization (HPF) have excellent performance during emulsion preparation even on an industrial scale, some studies have shown that treating protein with HPH and HPF directly at pre-emulsion stage could also contribute to a better interfacial property and emulsion quality (58–60). HPH provides both shear and cavitation forces to decrease particle size, changing structural properties and thus increasing emulsion capacity. Therefore, it is also widely used in improving emulsion properties of animal-derived protein including scallop protein (best at 100 MPa) (61), alkali-treated cod protein (best at 100 MPa) (62), TGase-induced whey protein isolate (best at 120 MPa) (63) and plant-derived protein like kidney bean proteins (best at 60 MPa) (41) and faba bean protein (decreased by HPH under 15 and 30 psi) (64). Many cases in globular proteins proved the HPH could facilitate the development of O/W interfaces because of forming soluble aggregates by either changing protein molecular conformation or disrupting the pre-formed large aggregates (41, 65). However, there is a study indicated that the soluble supramolecular aggregates induced by HPH might compete with native protein molecules at the interface, thus impair the quality of the interfacial network (64). Except for promoting soluble aggregates formation, HPH could also increase the migration rate and interfacial storage modulus of proteins by changing the tertiary structure and rearranging peptide chains (62). The result from Ali et al. (66) also indicated that since hydrophobicity of β-lactoglobulin was increased by HPH pretreatment, the adsorption rate and interfacial film formation of proteins are improved.

#### High intensity ultrasound

In food field, the low frequency (20–100 kHz) high intensity (10–1,000 W cm^–2^) ultrasound is recently employed for the modification of proteins (67). Previous work has reviewed that High intensity ultrasound (HIU) treatment is applicable in producing highly stabilized protein emulsions (67). The pretreatment of protein with HIU also potentially affects its emulsion abilities as determined by HIU mode, treatment period, ultrasound frequency, and intensity (68–70). Since the sonication might lead to highly exposed hydrophobic groups in proteins, it could effectively increase oil adsorption capacity and emulsion stability (69). It should be emphasized that HIU is often synergistically utilized with other treatments in improving emulsion properties of protein, such as HPH (63), amino acid fortification (70), and acid treatment (71). To be specific, combing HPH with HIU improved the interfacial properties of whey protein (improved by 13.97%), among which the former could solely increase the EAI by 8.54% (120 MPa) and the latter could solely enhance the EAI by 7.63% (600 W) (72). Similar to HPH, HIU-acid treatment improved the emulsifying properties of the soy protein isolate by dissociating aggregates, unfolding structure, and forming soluble aggregate (71). Also, through facilitating hydrophobic groups exposure and promoting more ordered structure formation, HIU-Arg addition contributed to myosin with better interfacial activity (70).

#### Other physical technologies

In addition to the promising tools discussed above, some other novel technologies, such as cold plasma and extrusion treatment, are also applied in improving the interfacial properties of proteins. Gong et al. (73) treated whey protein isolates with cold plasma and found that the interfacial elastic modulus was significantly strengthened for plasma-treated samples (10 s at 50 W power). Similar results on emulsion stability have been reported on peanut protein treated with dielectric barrier discharge cold plasma at 35 V and 2 A for 1, 2, 3, and 4 min (74). The enzymatic proteolysis of peanut protein isolate induced by extrusion pretreatment was able to reduce the saturated interfacial loads and enhance emulsion performance by hydrolyzing proteins into soluble fragments and generating surface active peptides (75). The extrusion cooking treatment is also applied in soy protein isolate modification. The larger aggregates formed by this treatment and the further disruption leaded by homogenization favored emulsion stability (76).

### Chemical technologies

#### Adjusting pH

The effects of pH on protein interfacial properties have been studied in many species, including faba bean (9), poppy seed protein (77), egg white/yolk protein (78), and whey protein (28). Under various pH values, the legume proteins suggested distinctive interfacial behavior since the surface charges, hydrophobicity and unfolding level were remarkably changed. It is concluded that pH has more influence on interfacial shear viscoelasticity than dilatational properties of protein. The effects of pH on protein emulsion changed depend on protein source. At a rather acid pH (i.e., 2.5 or 3.0), legume and poppy seed protein could form the strongest interface film and even with a faster rate, which might be induced by the more opened advanced structures (13, 77). Similarly, Tian et al. (79) found that β-conglycinin tended to form emulsions under acidic pH with decreased droplet diameters, leading to a faster diffusion rate and a higher ability for protein structural rearrangement compared with near isoelectric point. The stability of the Na-casein emulsion was maintained against coalescence for at least 1 month after lowering the pH to 1.8, which is likely attributed to the compaction of the monolayer and the increase in elasticity of the interfacial film (27). Whey protein exhibited increased interfacial stability at pH 9.0 than at pH 7.0 because of higher electrostatic repulsion and more hydrophobic structure formation (28). At approximately pI (pH 5.0–7.0), the egg yolk protein obtained a rather low ESI, which is attributed to the presence of insoluble yolk granular proteins of large size (78). Although many reports exhibited a better interfacial activity of protein at a rather extreme pH, it is also pointed out that at near pI conditions, proteins would form films with higher dilatational elasticity because of favored formation of multiple layers, shrinkage of hydrophilic segments, and higher intermolecular attractive interactions (80). Similar results were also reported in microalgae proteins; as the pH increased far from pI, the interfacial modulus decreased due to increased electrostatic repulsions between adsorbed proteins (81).

#### pH-shifting treatment

The pH-shifting process is also well-known as isoelectric solubilization/precipitation process, during which the alkalization/acidification and neutralization steps are commonly utilized to improve the interfacial and emulsion properties of many types of proteins derived from plants, milk, or eggs. Therein, the neutralization step is the main difference compared with the method of adjusting pH. Using alkali-heat treatment could, thus improving the flexibility of α-zein by deamidating, which resulted in higher amphiphilicity and emulsion ability (82). Researchers treated chia protein at pH 10 as well as 12 and recovered at pH 4.5, which profoundly affected the interfacial properties by changing structural properties of native protein (83). Acid treatment (pH 2.5) was proven to improve the adsorption rate to the O/W interface and allow the formation of a stronger interfacial film of soybean glycinin (84). For insoluble microalgae *Chlorella protothecoides* protein, acid-thermal hydrolyzed protein fragments could mix with protein aggregates in the interface, thus forming more stabilized layers (81). Jiang et al. (85) found that dephenol treatment could promote the improvement dominated by alkali shifting (pH 12.0) of canola protein isolates because of eliminating of endogenous phenolic hindrances, increasing conformational flexibility and surface activity, and improving dispersion.

#### Covalent bond formation on the interface

One of the most attractive topics is how the formation of protein-phenolic complexes improves the interfacial properties of proteins because of multifunctional effects (increased emulsifying ability, oxidation stability, and other bioactivities). This topic has also been fully comprehended by Farooq et al. (86). It is widely studied to produce phenolic-protein complexes to improve the initial interfacial activity of native whey, such as phytic acid (87), lotus seedpod proanthocyanidin (88), and cinnamaldehyde (89). Pei et al. (87) prepared an anionic phytic acid-whey protein complex and proved that the complex obtained higher cream resistance and a thicker cream layer than whey protein alone, which resulted from the faster adsorption rate, the higher ability to lower the interfacial tension and the increased electrostatic bridging between droplets. The covalent cross-linking formation at the O/W interface between whey and cinnamaldehyde favored the interfacial properties of the protein by promoting protein accumulation at the oil droplet surface (89). Felix et al. (90) proved a similar impact of cinnamaldehyde on protein isolates stabilized O/W interface, which is attributed to the increased homogeneity and surface rheological properties. Pan et al. (91) prepared chlorogenic acid-protein covalent complex under alkali conditions, resulting in higher adsorption ability onto the interface and more compact interfacial layers. However, for the flaxseed protein-phenolic complex system, complex formation could only strengthen the interfacial layer at low protein concentrations, while the complex exhibited a browning color because of alkaline-induced polymerization of phenolic compounds (92).

Enzyme catalyzation was also applied in improving protein emulsion properties by changing surface charges, increasing flexibility, modifying structure, or inducing self-assembly. A moderate TGase treatment (treated for 60 min) could improve the emulsion properties of faba bean protein isolates by increasing the net surface charge (93). Yu et al. (94) used TGase to cross-link Alcalase + trypsin hydrolyzed whey protein isolates, resulting in better emulsion properties, which is assumed to be due to increased flexibility and rearranged hydrophobic and hydrophilic amino acid residues. Solely hydrolyzed sodium caseinate by commercial enzymes could potentially self-assemble into network-like supramolecular particles, which drastically improved the stability of the emulsion by 400% (95). TGase also catalyzed glucosamine with soy protein, resulting in a stretched structure, softer state and higher flexibility compared to native soy protein isolates, thereby improving the ability to lower surface tension (31). HIU has been combined with dextran glycosylation to modify buckwheat protein isolates, which tended to be more closely packed and form thicker interfacial film (96).

#### Adding low molecular weight -surfactant

**FIGURE 2:**
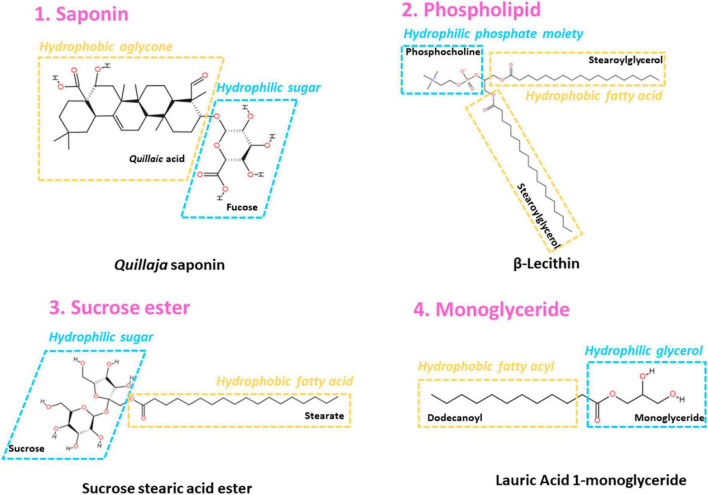
Representative low-molecular-weight surfactant structure mentioned in this work.

Generally, LMW surfactant is capable of displacing protein from the O/W droplet surface by an “orogenic” mechanism, thus undermining the stability of emulsions. However, at a proper ratio of surfactant to protein, the combination of these two could lead to a more compact interfacial layer (97). Therefore, the impacts of LMW surfactant on the interfacial properties of proteins are controversial, and it is found to be related to the native structure, concentration and types of both surfactant and polymers. The LMW surfactant consists of a hydrophilic head and one or several hydrophobic tails. Directly adding natural LMW surfactants such as saponins (98), phospholipids (99), sucrose esters (100), monoglycerides (101), and small peptides (102) could enhance the interfacial properties of proteins. The interfacial activity of these LMW surfactants differs because of various structural features (Figure 2):

• *Saponins*: Some saponins consist of a hydrophobic aglycone structure and hydrophilic sugar residues, and the adjacent sugar residues in the interface could form hydrogen bonds to stabilize an interfacial film Böttcher et al. (103).
• *Phospholipid*: Some phospholipids show high surface activity due to the hydrophilic phosphate moiety head and hydrophobic fatty acid tail (6).
• *Sucrose ester*: Sucrose fatty acid ester is commonly used as a non-ionic surfactant because of the contribution of both the hydrophilic sucrose head and the hydrophobic fatty acid tail (100).
• *Monoglyceride*: Monoglyceride could act as an emulsifier in the food industry due to the hydrophilic glycerol backbone attached to the hydrophobic fatty acyl chain (104).
• *Small peptide*: To achieve better emulsifying activity, the small peptide emulsifier should exhibit amphiphilic properties by consisting of a hydrophobic and a hydrophilic region independent of the secondary structure, and these regions better show an axial state (105).

Soy saponin addition loosened the rigid conformation of the protein and improved resilience to external deformation of both β-conglycinin (7S) and glycinin (11S), which further led to long-term stability (up to 42 days) (98, 106). Böttcher et al. (103) demonstrated that saponin-β-lactoglobulin complex formation, which is mainly governed by hydrogen bonds and/or hydrophobic interactions, contributing to a stronger and higher viscoelastic interface. A study performed previously applied HIU to produce chickpea protein and ginseng saponin complex, which significantly increased interfacial adsorption and smaller droplets compared to solely protein molecules (107). However, by studying the interfacial co-adsorption aging of *Quillaja* saponin with pea protein, it is suggested that the mixed saponin-protein interface exhibited a more fluid-like behavior instead of an elastic one, indicating that the effects are highly dependent on the type of complex formed (8).

Adding a low level of lecithin (0.5–1.0%) would help to decrease the O/W interfacial tension of mussel water-soluble protein, while a high level of lecithin (1.5–2.0%) led to unfavorable protein aggregation and unfolding because of competitive adsorption (99). Wang et al. (108) also indicated that soy lecithin affected emulsions stabilized by whey protein isolate in a concentration-dependent manner, among which a low lecithin concentration (0.25–0.75%) endowed better emulsion formation by synergistically improving interfacial properties (e.g., making a rather rigid interfacial film). Lecithin (final concentration of 2%) also synergistic with gelatin in solubilizing oil-loaded emulsions (109).

The competition behavior induced by monoglycerides is determined by both their content and protein subunits. Li et al. (110) indicated that the addition of monoglycerides affected the network formation of β-conglycinin in a synergistic or competitive adsorption manner when the protein concentration was low (4%) or high (>6%), respectively, as reflected by drastically changes in the interfacial elastic modulus G’. Additionally, 0.1 wt% monoglyceride is capable of displacing acid and basic soy protein subunits from the interface, thus reducing the amount of adsorbed protein but not β-subunits (45).

To be addressed, bioinformatics tools have been extensively utilized in predicting the emulsion properties of peptides from rich resources. Hydrolysed peptides are derived from potato (105), soy (111), and seaweed (112). As predicted, the peptide had the highest amphiphilic score, and proper length obtained the best interfacial and emulsifying activity. Peptide could act as emulsifier in two modes: constituting inner interfacial membrane through partitioning and weakly bonding to inner interface and forming a surrounding external layer (113). Introducing amphiphilic peptides into protein systems or simply hydrolyzing proteins to a certain level could change their interfacial and emulsion activities. It has been reported that the addition of zein hydrolysate effectively improved the emulsifying stability of myofibrillar protein by helping to form a more compact and massive interfacial membrane (102). However, another study proved that hydrolyzed peptides showed a disadvantageous impact on EAI and ESI of sunflower protein, which was likely due to the lower ability of peptides to reduce interfacial tension (114).

Compared to the above LMW emulsifiers, some other types are also noticed with limited attention. Zhao et al. (100) studied the effects of the content of sucrose ester on the interfacial properties of sodium caseinate. A lower concentration (<0.05%) led to co-adsorption at the interface, while a higher concentration (>0.05%) of the interfacial interactions between sodium caseinate and sucrose ester governed competitive adsorption. Although BSA (66 KDa) and lysozyme (14.3 KDa) are both globular proteins, the combination of ionic LMW surfactant [C12mim] Br showed co-adsorption behavior on lysozyme but competitive adsorption on BSA, which indicated that the function of LMW surfactant also varies depending on molecular weight (115).

#### Other chemical technologies

In contrast to covalently bonded protein-phenolic conjugates, some studies have focused on the effects of non-covalently bonded protein-phenol complexes on the interfacial properties of proteins. It has been reported that sunflower protein-chlorogenic acid complex formation, which is mainly driven by hydrogen bonds, resulted in a higher ability to decrease interfacial tension than protein formation alone (116). The addition of alkaline amino acids, such as arginine and lysine, was reported to facilitate many functionalities of proteins, including interfacial activity and thus emulsion properties. Both arginine and lysine increased the adsorbed protein and penetration rate but decreased the diffusion rate of interfacial pork myosin, leading to improved EAI and ESI (49). A higher level of phosphorylation resulted in a reduction in particle size and loosening of the protein structure because of interactions between phosphate groups and amino acid residues, therefore improving the emulsion ability (30).

## Potential mechanisms upon improving interfacial properties

Applying these above treatments to modify protein structure and physicochemical properties effectively improved interfacial properties through the following pathways, as illustrated in Figure 1:

*(1) Aggregates formation*: a. Thermal treatment could enhance protein aggregation to a larger size. Although the increased molecular size potentially lowered the diffusion and rearrangement rate, it also allowed larger steric hindrance formation at the droplet surface, exhibiting higher viscoelasticity. b. Treatments such as HPH and HIU led to soluble aggregate formation by cavitation and turbulence functions, which contributed to better interfacial film formation; *(2) Reduced molecule size:* A smaller sized-protein polymer could be more easily adsorbed onto the O/W interface with a higher migration and diffusion rate, which often results from HPH and HIU enhancement.

The faster speed would let oil droplets stabilize before destabilization, such as coalescence and flocculation; *(3) Amphiphilicity modification:* Many treatments are reported to be able to modify advanced structures, especially leading to hydrophobic group exposure, such as HPT, pH shifting, and TGase catalysis. A more balanced polar and nonpolar group distribution surely favors interfacial adsorption and film formation; *(4) Increasing flexibility:* Higher flexibility contributed to faster penetration and rearrangement rates, which led to better interfacial properties. The relatively easier way to adjust the flexibility of proteins is to change the pH. The altered electrostatic repulsion force would tune flexibility around a certain range. In addition, HPT could drive proteins to form a molten globule state, thus increasing flexibility. Glycosylation and phosphorylation were also reported to promote flexibility by introducing new groups; *(5) Co-adsorption and stabilization*: Although natural LMW surfactants may displace proteins from the interface, under proper conditions, they can synergistically lower the interfacial tension and guarantee the stability of surface layer-covered droplets; *(6) Facilitating interfacial protein interactions:* One of the main benefits of increasing the surface hydrophobicity of proteins is strengthening protein–protein interactions and forming more compact and elastic networks at the interface. Phenolic compounds grafting also attained similar results because of phenolic-mediated cross-links.

To be emphasized, inappropriate or excessive parameters of such treatments could also deteriorate interfacial activity by forming a softer interfacial film, aggregating to retard diffusion or penetration rate, impeding structure stretching after adsorption, and competitively adsorbing to the interface. Therefore, proper processing conditions are inevitable for effectively facilitating protein interfacial properties. Since each single treatment has been more deeply revealed for decades, a trend focused on associating two or even more treatments to spontaneously enhance interfacial activity and thus emulsion properties is gradually attracting more attention. For example, HIU treatment is suitable to be combined with catalysis and hydrolysis processes to yield synergistic results. pH-shifting treatment is always followed by heating to produce fiber- or particle-like protein agglomerations and change their interfacial behavior. At the end of this review, we address the fact that since the relationship between emulsion properties and interfacial behavior is not considered simply straightforward, the correlations between these two functionalities need to be more clearly elucidated.

## Conclusion and future trends

In this review, the key natural characteristics of proteins in determining interfacial behaviors and properties were firstly summarized, and their effects on emulsion properties were discussed. Secondly, the improving strategies tailored to improving the interfacial activity of proteins were reviewed in two subsections according to distinctive mechanisms: physical treatments (mainly including thermal treatment, high-pressure treatment, high-pressure homogenization, and high-intensity ultrasound) and chemical modifications (mainly including pH adjustment, covalent modification, and LMW surfactant combinations).

Although the interfacial proteins have been attracted attentions and widely studied, most of researches are prone to delineating the changes of structural and emulsifying properties of these proteins. Mainly processing properties are focused and deeply revealed. However, as the topics caring food nutrition and human health are urgently needed, a lot of work could be conducted at this field. We have proposed some hot topics related to interfacial protein film following:

(1) Relation between interfacial protein and proteins in continuous phase should be more addressed, especially in meat protein system. In meat protein field, since the emulsion product is not typical O/W emulsion, the quality of emulsified meat is not only dependent on inner interfacial film properties (whether they are regular, dense, or compact), but also largely decided by the interaction between interface and sub-interface layer and also the rigidity as well as strength if covered oil droplets. Many works required to be conducted to better unveil the mechanism regarding this aspect.

(2) As bioinformatics and *in silico* tools like molecular dynamics simulation are rapidly developed, more computational investigation toward interfacial behavior of proteins should be performed. Based on these novel approaches, more detailed information about molecular forces and steric hindrance impact of proteins will be clarified and the interfacial behavior of a single protein molecule could be elucidated.

(3) It is still remained unknown that how does the microscopic interfacial properties give light on upgrading of large-scale industrial processing. To achieve this goal, diversity of the protein species including globular, flexible, and fiber-like molecule, should be implemented in measuring interfacial stage behavior and emulsion analysis, such as emulsion stability, emulsion activity, and emulsion-gel quality. These databases could provide a platform to highlight the practical meaning of interfacial properties.

(4) Although many scientists have already noticed the interfacial region was closely related with flavor release, bioactive protector, and gastrointestinal behavior of emulsions, the information of oral taste, digestion and bioactive delivery properties of O/W emulsions as a function of protein interfacial behavior is still lacking. Therefore, extra attention should also be paid to interpreting how interfacial properties influence sensory perception and human acceptance by altering emulsion rheological properties, oral processing behavior and product texture. Also, the relationships between interfacial properties and digestive and bioaccessibility characteristics also urgently need to be fully understood.

## Author contributions

HZ: investigation-equal, validation-equal, visualization- equal, and writing—original draft-equal. XZ: conceptualization-lead, funding acquisition-lead, project administration-lead, resources-lead, supervision-lead, writing—original draft-equal, and writing—review and editing-lead. XC: administration-supporting and supervision-supporting. XX: conceptualization-supporting, funding acquisition-supporting, project administration-supporting, and supervision-supporting. All authors contributed to the article and approved the submitted version.
